# Growth Kinetics of Probiotic *Lactobacillus* Strains in the Alternative, Cost-Efficient Semi-Solid Fermentation Medium

**DOI:** 10.3390/biology9120423

**Published:** 2020-11-27

**Authors:** Katarzyna Śliżewska, Agnieszka Chlebicz-Wójcik

**Affiliations:** Institute of Fermentation Technology and Microbiology, Faculty of Biotechnology and Food Sciences, Lodz University of Technology, Wólczańska 171/173, 90−924 Łódź, Poland

**Keywords:** *Lactobacillus*, cultivation, flours

## Abstract

**Simple Summary:**

Commercial microbiological media are often expensive because of used ingredients; therefore, their application in high-density cell production is cost-consuming. Since *Lactobacillus* bacteria are commonly used in various industries, and especially as probiotics, alternative cheaper media are needed. Additionally, the approach is to use food or agriculture by-products, which can support the growth of bacteria due to their components. Among them, cereals and flours are used worldwide; therefore, their usage as a medium for *Lactobacillus* spp. can be accessible, easy, and affordable. Results obtained from the following study showed that a mixture of wheat, barley, maize, and rye flours combined with distilled water is a good medium for the efficient growth of selected probiotic *Lactobacillus* spp.

**Abstract:**

The growing need for *Lactobacillus* bacteria usage in industry and the expending probiotic market led to a search for new cost-efficient fermentation media from which a high yield of these bacteria could be obtained. The following study aimed to elaborate cultivation medium, for *Lactobacillus* spp. growth, which main components would be wheat, maize, barley, and rye flours. The optimal temperature for *Lactobacillus* growth in new semi-solid fermentation (SSF) medium, water content, and pH of the medium were analyzed by the plate count method. It was established, that the highest bacteria counts were obtained from cultures conducted in the SSF medium with flours to water ratio of 1:1.5 with a natural pH of 6.0 at 37 °C. Subsequently, the growth kinetics of analyzed strains, in both MRS and the SSF media, were studied. The newly designed media contributed to the increased duration of selected Lactobacillus strains lag phase, which varied from 1.98 to 5.64; nevertheless, the maximum growth rate of the strains was two times higher in the SSF medium rather than in MRS, which also resulted in shorter generation time. The developed medium has the potential to become a new cost-efficient fermentation medium for *Lactobacillus* spp.

## 1. Introduction

Species belonging to *Lactobacillus* genera are ones of the most wildly used probiotics in animal feeding, which are defined as live microorganisms conferring health benefits to a host when administrated in the proper dosage [1,2]. Probiotics are proved to have an advantageous effect on people suffering from gastrointestinal system disorders, such as infectious diarrhea, inflammatory bowel diseases, celiac disease, and food allergies among many others [3]. What is more, Cicero et al. (2020) recently described the positive effect of *Lactobacillus* spp. probiotic strains, in the presence of inulin and fructooligosaccharides, on elderly patients, whose cardiovascular and insulin resistance risk factors, related to metabolic syndrome, were decreased [4]. Moreover, these beneficial microorganisms are used as an alternative for antibiotic growth promoters (AGPs), which were prohibited in European Union (EU) in 2006, which caused an increase in the infection rate among livestock [2]. The probiotic market is foreseen to bring 74 billion USD income in 2024, which is over two times higher than in 2015 (35 billion USD) [5].

The growing need for *Lactobacillus* spp. usage in the industry resulted in the necessity of obtaining a high density of these bacteria at low cultivation costs [6,7]. *Lactobacillus* spp. are characterized by high nutritional requirements, due to their weak ability to synthesize amino acids and vitamins from B-group [8]. Therefore, their cultivation needs to be conducted in a rich medium, which includes fermentable carbohydrates, nucleic and amino acids, B-complex vitamins, as well as different minerals [9]. Moreover, *Lactobacillus* spp. bacteria primarily use peptides in order to fulfill their demand for nitrogen [10]. For LAB cultivation purpose, on a laboratory scale, meat or yeast extract are commonly used as a nitrogen source; however, these components contribute to the high cost of ordinarily used de Man, Rogosa, and Sharpe medium (MRS) [11]. Low-cost medium alternatives are looked for mainly among food and agriculture by-products and wastes [10]. Cereals such as wheat, barley, maize, or rye, which are commonly used for animal feed, are proven to be a good source of nutrients for many LAB species [9,12].

Besides medium composition, *Lactobacillus* spp. growth can be influenced by a set of various conditions such as temperature, pH, oxygen concentration, or water activity [13,14]. The optimum temperature and pH conditions for lactobacilli growth are 30–40 °C and 5.5–6.2, respectively; however, the *Lactobacillus* genus is diversified and belonging bacteria can grow in temperature ranging from 2 to 53 °C and pH varying between 4.5 and 6.5, some strains can grow in even lower pH [15]. Culture conditions as well as fermentation medium composition, in which *Lactobacillus* spp. bacteria are grown, can influence growth kinetics parameters such as specific growth rate and lag phase duration, which is the time in which bacteria adapted to new media and do not proliferate [16,17,18].

The study aimed to develop a new cost-efficient medium based on commercially available agriculture products used in monogastric animals feeding, such as cereals-derived flours, for *Lactobacillus* spp. cultivation. The scope of the research consisted of the determination of optimal growth conditions, namely, water content and pH of the designed medium as well as temperature. Moreover, the growth kinetics in both MRS and newly elaborated medium were estimated and compared.

## 2. Materials and Methods

### 2.1. Strains

Subjects of the research were *Lactobacillus* strains with documented probiotic properties, namely *Lb. paracasei* ŁOCK 1091, *Lb. pentosus* ŁOCK 1094, *Lb. plantarum* ŁOCK 0860, *Lb. reuteri* ŁOCK 1092 and *Lb. rhamnosus* ŁOCK 1087 [19,20,21,22,23,24]. For the strains isolation from alternative sources, viz the gastrointestinal tract (GIT) of monogastric animals, such as broiler chickens and pigs, as well as plant silage, the Rogosa agar (BD Difco™, Sparks, NV, USA) was used. The *Lactobacillus* strains were deposited in the Lodz Collection of Pure Cultures (ŁOCK 105) of the Institute of Fermentation Technology and Microbiology (Lodz University of Technology; Lodz, Poland) and their beneficial impact on monogastric animals were previously analyzed [25,26,27].

For the storage purposes of *Lactobacillus* spp. the Cryobanks™ (Copan Diagnostics Inc., Murrieta, CA, USA) were used, which were kept at −22 °C.

### 2.2. Inoculum Preparation

The strains were activated and then passaged twice in de Man, Rogosa, and Sharpe broth (MRS, pH = 6.2 ± 0.2; Merck Millipore, Burlington, MA, USA). The incubation of the strains was conducted at 37 °C for 24 h. Subsequently, the strains were centrifuged at 10,732× *g* relative centrifugal force (RCF) for 10 min (Centrifuge MPW-352; MPW, Warsaw, Poland). The obtained biomass of each strain was washed with 0.1 M phosphate-buffered saline (PBS; Calbiochem^®^, Merck Millipore, Burlington, MA, USA) by centrifuging. Afterward, the cells were resuspended in the PBS and optical density (OD) was adjusted to correspond to 10^7^ colony-forming units per milliliter (CFU/mL) cell density at 600 nm wavelength (Beckman DU 640, Beckman Coulter Inc., West Sacramento, CA, USA).

### 2.3. Semi-Solid Fermentation Medium Design

Based on the natural flours commonly used in livestock feeding, such as wheat (40% *w*/*w*; Młyny Szczepanki Sp. z o.o., Łasin, Poland), barley (30% *w*/*w*; Młyn Oliwski, Gdańsk, Poland), maize (20% *w*/*w*; RADIX-BIS Sp. z o.o., Rotmanka, Poland) and rye (10% *w*/*w*; Polskie Młyny S.A., Warsaw, Poland) flours, the semi-solid fermentation (SSF) medium was designed (pH = 6.0 ± 0.2). The medium composition was determined on the basis of the literature review. Flours nutritional values, declared by producers, are presented in Table 1.

Distilled water was added to the flours mixture in three different ratios, namely 1:1, 1:1.5, and 1:2. Subsequently, the SSF media were sterilized by autoclaving and inoculated with the *Lactobacillus* strains separately. After 24 h of incubation at 37 °C, 1 g of sample was suspended in 10 mL sterile saline solution, homogenized, and serial decimal dilutions were prepared. The bacteria number was determined by the plate count method with the use of MRS agar. Each experiment was conducted in three replication and the bacterial count was given as CFU per gram (CFU/g). Moreover, viable cells productivity (VCP) was calculated (1) according to Ming et al. (2016) with modifications [28]:

(1) $$VCP=\;\frac{X}{t},$$

where *X* stands for total viable cell count (CFU/kg), whereas *t* is the total fermentation time (h).

### 2.4. The Effect of Temperature and pH on the Growth of Lactobacillus pp. Strains

Each strain’s inoculum was introduced into tubes with MRS broth or SSF medium with standardized pH of 6.2 ± 0.2, as well as with modified initial pH set at 4.0, 4.5, 5.0, 5.5, 6.0, and 6.5. To establish the temperature impact on the strains’ growth, the tubes with inoculated media with standard pH were incubated at 4, 20, 30, 37, 44, and 55 °C for 24 h. The pH level effect on the growth of the strains was determined after cultivation at 37 °C for 24 h.

After the incubation process, serial decimal dilutions were prepared and the number of cells was detected by the plate count method with the usage of MRS agar. Before dilution, 1 g of each sample obtained from the SSF medium was suspended in sterile saline solution (10 mL) and homogenized. Next, plates were incubated at 37 °C for 48 h and, subsequently, the bacterial count was given as CFU/g or CFU/mL. Each parameter for all the strains was tested in three replications.

### 2.5. Growth Kinetics of the Lactobacillus Strains in the Semi-Solid Fermentation Medium

The probiotic strains were cultivated in the SSF medium, and MRS broth comparatively, for 30 h in the temperature and pH conditions adjusted accordingly to the obtained data from point 2.4. The number of bacteria was established analogous as in point 2.4, at the time of inoculation and after 4, 8, 12, 16, 24, and 30 h of incubation. Moreover, the pH of the culture media was measured at the same time points. The assay was performed in triplicate.

Estimation of the growth curve was performed with the Gompertz model in the Origin 6.1 software (OriginLab Corp., Northampton, MA, USA). DMFit version 3.5 (ComBase, https://www.combase.cc/), which is an Excel add-in, was used to establish maximum growth rate (µmax) and duration of lag phase (λ), as well as, to calculate a generation time (GT) for every strain cultivated in each medium.

### 2.6. Statistical Analysis

Statistical analysis was performed with XLSTAT software (Addinsoft, SARL, Paris, France). The data showed herein constitute the arithmetic means of values from three repetitions. One-way analysis of variances (ANOVA), at a significance level of *p* < 0.05, was performed for outcomes obtained from growth performance assay in SSF media with different water contents, as well as for VCP results. Significantly different mean values were marked with various lowercase letters (a—c). Before performing ANOVA, data were checked for normal distribution (Shapiro-Wilk test) and homogeneity of variances (Bartlett’s test). Moreover, Tukey’s post hoc test was used after each ANOVA.

Furthermore, heatmaps were used to present outcomes gathered from growth performance assay in SSF medium in various temperature and pH conditions. Plots were prepared with the usage of the ClustVis web tool (https://biit.cs.ut.ee/clustvis/), data were not scaled or transformed. Additionally, the hierarchical clustering analysis (HCA), based on Euclidean distance and average linkage, was applied.

## 3. Results

### 3.1. Effect of the Water Content in Semi-Solid Fermentation Medium on the Growth of Lactobacillus Strains

**Figure 1:**
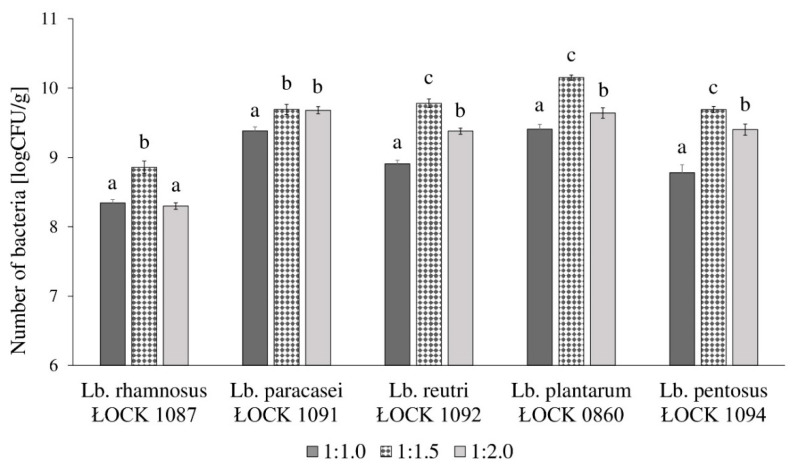
Growth performance of five *Lactobacillus* spp. (abbreviated as *Lb.*) in the semi-solid fermentation (SSF) medium, with three different water contents, in which the ratio of flours to water was as follows: 1:1.0, 1:1.5, and 1:2.0. ŁOCK is a designation of strains obtained from the Lodz Collection of Pure Cultures (Institute of Fermentation Technology and Microbiology, Lodz University of Technology; Lodz, Poland). Moreover, different lowercase letters (a–c) stands for significantly various mean values of bacterial growth per one strain according to performed one-way ANOVA with post hoc Tukey’s test (*p* > 0.05).

The highest growth of selected *Lactobacillus* spp. was observed when bacteria were cultivated in the SSF medium with flours to water ratio of 1:1.5. The number of *Lactobacillus* spp. varied between 8.86 and 10.15 log(CFU/g). Except for *Lb. paracasei* ŁOCK 1091 strain, the higher water content in the SSF medium resulted in significantly weaker growth and the average number of bacteria was 9.28 log(CFU/g). The lowest bacteria count was obtained when strains were cultivated in a medium with flours to water ratio of 1:1.0. The results are presented in Figure 1.

The VCP was contingent on the water content in the newly designed SSF medium (Table 2). An increase of flours to water ratio, from 1:1.0 to 1:1.5, resulted in up to 7.5 times higher VCP. Nonetheless, a further increase in water content (ratio: 1:2.0) in the SSF medium diminished the VCP, which varied from 9.44 × 10^9^ to 1.85 × 10^11^ CFU/(kg × h). This indicates a decrease in the productivity of between 2.68 and 69.11% when compared to the same parameter obtained in the medium with flours to water ratio of 1:1.5. However, the VCP in SSF medium with flours to water ratio 1:2.0 was up to almost 4 times higher than in the same medium with water content equal to the amount of the flours (ratio: 1:1.0).

### 3.2. Temperature and pH Levels Impact the Growth of the Selected Lactobacillus Strains

**Figure 2:**
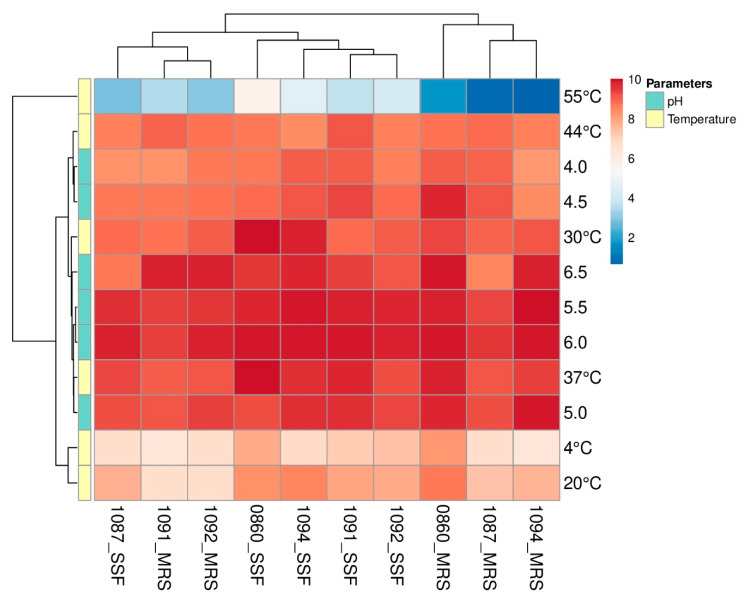
A heatmap presents the growth of analyzed *Lactobacillus* spp. (*rhamnosus* ŁOCK 1087, *paracasei* ŁOCK 1091, *reuteri* ŁOCK 1092, *plantarum* ŁOCK 0860 and *pentosus* ŁOCK 1094) which were cultivated in de Man, Rogosa, and Sharpe (MRS) broth or the semi-solid fermentation (SSF) medium, with standardized pH, in diverse temperatures, or various pH levels of media in 37 °C. The obtained numbers of bacteria are displayed as the logarithm of colony-forming units per milliliter log(CFU/mL) (MRS medium) or gram log(CFU/g) (SSF medium). Strains are listed at the bottom of the heatmap as the ŁOCK collection numbers with the medium in which they were cultivated, whereas modified growth parameters are given on the right side of the plot.

Selected *Lactobacillus* strains showed the ability to adapt and grow in a wide spectrum of temperatures and under varied pH conditions (Figure 2). Based on the horizontal dendrograms presented in Figure 2, the highest count of bacteria, regardless of used media, was observed when cultures were conducted at 37 °C and the initial pH of the medium was varying from 5.0 to 6.0. Nevertheless, the substation of MRS with the SSF medium resulted in enhanced growth of *Lb. plantarum* ŁOCK 0860 and *Lb. pentosus* ŁOCK 1094 at 30 °C. Furthermore, the heatmap shows that the highest number of *Lactobacillus* spp. was obtained in the SSF medium with an initial pH set at 6.0. On the other hand, the initial pH of MRS that resulted in the highest yield of cells was strain-dependent and varied from 5.5 to 6.5. The optimal growth conditions (37 °C, pH = 6.0) resulted in a better proliferation of *Lactobacillus* strains in the SSF medium than in MRS, which is shown by vertical dendrogram indicates (Figure 2).

### 3.3. The Growth Kinetics of the Lactobacillus Strains in the Semi-Solid Fermentation Medium

**Figure 3:**
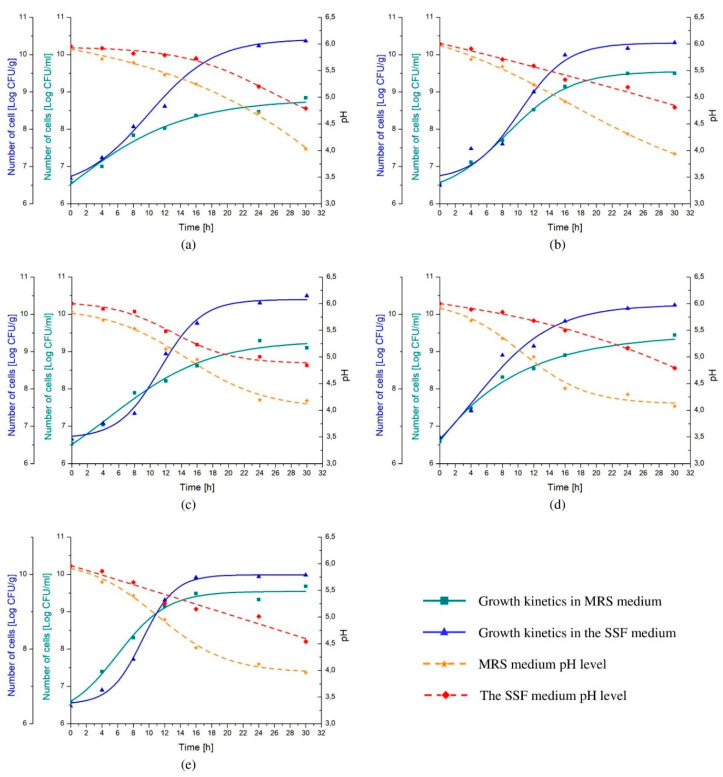
The growth kinetics of selected *Lactobacillus* spp., namely, *Lb. rhamnousus* ŁOCK 1087 (**a**), *Lb. paracasei* ŁOCK 1091 (**b**), *Lb. reuteri* ŁOCK 1092 (**c**), *Lb. plantarum* ŁOCK 0860 (**d**) and *Lb. pentosus* ŁOCK 1094 (**e**) cultivated in the semi-solid fermentation (SSF) medium or de Man, Rogosa, and Sharpe (MRS) broth, comparatively. Results are presented as the logarithm of colony-forming units per gram (Log CFU/g) or milliliter (Log CFU/mL).

Higher numbers of cells were reached when each of the analyzed probiotic strains was cultivated in the SSF medium rather than in commercially available MRS (Figure 3). After 30 h of incubation, cell density obtained from *Lactobacillus* spp. cultures conducted in MRS varied between 8.85 ± 0.19 and 9.68 ± 0.08 log(CFU/mL), whereas when probiotic monocultures were grown in the SSF it was higher by up to 1.5 decimal logarithmic unit (the average of 10.28 log(CFU/g)). On the other hand, pH level decreased more substantially in MRS than in the SSF medium, reaching value ranging from 3.94 ± 0.15 to 4.18 ± 0.19 and from 4.54 ± 0.32 to 4.84 ± 0.17, respectively, subsequently to 30 h of cultivation (Figure 3).

Moreover, the cultivation of selected *Lactobacillus* spp. in the SSF medium resulted in up to two times higher maximum growth rate (μ_max_), which varied from 0.21 to 0.37 h^−1^, whereas µ_max_ of bacteria grown in MRS medium did not exceed 0.23 h^−1^ (Table 3). Furthermore, the generation time (GT) was shorter when the SSF medium was used instead of MRS for the growth purposes varying from 0.96 to 1.41 h and 1.32 to 2.61 h, respectively. Nevertheless, the tangible lag phase (λ) was observed in the *Lactobacillus* strains cultures in the SSF medium reaching up to 5.64 h, with exception of *Lb. plantarum* ŁOCK 0860 (Figure 3, Table 3). Contrary, a clear adaptation stage in the MRS medium was observed only when *Lb. paracasei* ŁOCK 1091 was inoculated.

## 4. Discussion

Cereals are mainly composed of carbohydrates, which constitute up to 75% of a grains dray mass; however, they are also a good source of proteins, whereas lipids are a minor part of their nutrients (1.7 to 7.0% of dry mass) [29]. Moreover, grains contain a considerable amount of vitamin E and ones belonging to the B-group as well as minerals such as zin, ion, magnesium, and calcium, which are necessary for *Lactobacillus* spp. growth [29,30]. Despite the substantial reduction of the nutritional value of cereals during the process of milling, which is defined as grinding grains into flour or meal, their bioavailability is increased [31]. On this basis, the flours were chosen as the main components of the SSF medium used for selected *Lactobacillus* strains in the following study.

Water activity and pH level of cultivation medium, as well as the temperature of incubation, are one of the most influential factors in terms of growth, viability, and activity of probiotic cultures [32]. It was established that the highest numbers of each *Lactobacillus* strain, up to 10^10^ CFU/g, were obtained in the medium combining flour and water with a ratio of 1:1.5, respectively. Denkova and Krastnowa (2012) observed a slightly higher count of *Lactobacillus* spp., even up to 10^11^ CFU/g, in the medium in which the ratio of wheat flour to water was 44%:56% [33]. However, on the contrary to our research, analyzed *Lactobacillus* strains were isolated from the naturally fermented sourdough, which may result in their better ability to colonize the environment than strains, described in our paper, isolated from livestock’s GIT or plant silage [34].

To the current day studies of flours fermentation by lactic acid bacteria were mostly focused on these microorganisms’ impact on sourdough features and bread-making, rather than the cultivation of bacteria and optimal growth conditions. Therefore, outcomes obtained from this study could be compared only with data gathered by researchers who performed analysis in other cultivation media. Sánchez et al. (2019), who reviewed over 30 researches considering various *Lactobacillus* strains growth requirements and conditions, observed that the incubation temperature of these bacteria varied from 23 to 42 °C, whereas pH ranged between 5.2 to 6.9, depending on the used fermentation media [35]. In the following research. growth of analyzed *Lactobacillus* strains was observed in a wide range of temperatures (20–44 °C) in both studied media; however, the highest yields of cells were obtained when the strains were cultivated at 37 °C, which was in line with outcomes gathered by Lim (2010) [36]. Yang et al. (2018) also noted that the temperature of cultivation is influential in terms of *Lactobacillus* spp. growth and regardless of used medium, namely, MRS or Brain Heart Infusion broth (BHI), the highest bacteria population density was observed at 37 °C, which was in agreement with data presented in the following paper [37]. Furthermore, the analyzed *Lactobacillus* spp. also adapted well to the various initial pH levels, ranging from 4.0 to 6.5, regardless of used media, which was in line with the results described by Lim (2010) [36]. In the SSF medium with pH value set at 6.0, all of the analyzed strains obtained the highest numbers of cells. The natural pH of used flours, namely, wheat, barley, maize, and rye, oscillates mostly around 6.0, and after they were mixed with water the SSF medium initial value was exactly 6.0 without any adjustments [38,39,40]. These indicate that there might not be needed substantial pH alterations in the SSF medium, which could reduce the costs of medium production. On the other hand, the impact of the initial pH of the MRS was strain-dependent and the optimal value ranged between 5.5 and 6.5, which could raise the difficulty in adjusting the initial pH for the production of all strains. Juarez Tomás et al. (2002) noted, that the optimal initial pH for *Lb. salivarius* subsp. *salivarius* CRL 1328 was 6.5, despite used media, namely, MRS or LAPTg broths [41]. Meena et al. (2014), however, observed the growth of *Lb. acidophilus* NCDC 14 in MRS with initial pH ranging from approximately 3.0 up to more than 7.0, whereas the optimal initial pH was 6.08. These differences in optimal initial pH levels, established by other research teams, underline the strain-dependency in growth performance in MRS broth regarding initial pH, which was not observed for the SSF medium in the following research [42].

The growth kinetics analysis of each strain was performed in the previously determined optimal conditions of temperature (37 °C) and pH (6.0), in both MRS broth and the newly developed SSF medium. A substantially higher number of each *Lactobacillus* strain was observed in the SSF medium; however, on the contrary to the cultures conducted in MRS, strains monocultures, except *Lb. plantarum* ŁOCK 0860, exhibited unequivocal adaptation phase (lag phase), which average duration was 4.10 ± 1.56 h in the alternative medium. Herrera--Ponce et al. (2014) observed comparable lag phase duration when monocultures of *Lb. casei* (431) and *Lb. acidophilus* (LA-5^®^) were cultivated in a simple oat medium (oat flour mixed with tap water) [43]. Nevertheless, preadaptation of selected *Lactobacillus* strains to the SSF medium could result in a reduction of lag phase duration, since genetic changes might need to occur for strains to be able to adapt its metabolism and grow in the new media [17,44]. Despite the elongated lag phase, not only cell yields were increased but also up to more than two times higher growth rates were noted during the cultivation of analyzed strains in the SSF medium. These findings were in agreement with Brignone et al. (2017), whose research aimed to select a substance for *Lactobacillus* spp. growth enhancement [45]. Moreover, comparable growth rates, as one exhibited by *Lb. rhamnosus* ŁOCK 1087 (0.21 h^−1^) and *Lb. plantarum* ŁOCK 0860 (0.22 h^−1^), were observed by Saman et al. (2011), who analyzed *Lb. plantarum* NCIMB 8826 growth in whole brown rice and rice bran broths [46]. The growth of the rest of the analyzed strains was faster, and its rate varied from 0.27 to 0.37 h^−1^, which was similar to the results obtained by Ruiz Rodríguez et al. (2019), who cultivated *Lactobacillus* spp. strains in formulated fruit simulation medium (FSM) [47]. These indicate the dependency of growth rate not only on medium composition but also on strains. Furthermore, the generation time was also in relation to strain and media used for its cultivation. The shortest doubling time was observed for *Lb. pentosus* ŁOCK 1094, whereas the longest time to double the number of cells was needed for *Lb. rhamnosus* ŁOCK 1087, in both MRS broth and the SSF medium. Nevertheless, it was noted that the replacement of MRS broth with tested SSF medium resulted in up to over 2 times shorter generation time, which oscillated around 67.80 ± 15.54 min. Similar results were obtained by Bundinich et al. (2011), whose research team cultivated *Lb paracasei* ATCC 334 in cheese extract medium obtained from Cheddar cheese of different age; however, the final number of cells was significantly lower than observed in the following analysis [48]. In summary, the results of the performed growth kinetics analysis show a positive effect of the newly developed SSF medium usage, even despite the elongated lag phase.

The shift from the MRS broth to the SFF medium as the cultivation environment not only impacted the growth kinetics of selected *Lactobacillus* strains, but also the changes in media pH levels. The decrease of MRS broth pH values during incubation of selected strains monocultures was more considerable than observed in the SSF medium. The final average pH level of MRS broth was 4.04 ± 0.10, which was in line with the results described by Shokryazdan et al. (2015), whereas the SSF medium mean pH, after 30 h of *Lactobacillus* spp. growth, was 4.75 ± 0.12 [49]. The end products of lactic acid bacteria fermentation, mainly organic acids, such as lactate or acetate, which are associated with the antimicrobial activity of strains, are responsible for the reduction of medium pH levels [50,51,52]. Zalán et al. (2010) previously observed that medium composition influences organic acids production [53]. Moreover, Śliżewska and Chlebicz-Wójcik (2020) noted significant variations in metabolic profiles of analyzed *Lactobacillus* spp., especially organic acids production, when strains were cultivated in MRS broth with different carbon source [54]. Therefore, one of the reasons for the limited reduction of the SSF medium pH could be decreased synthesis of organic acids caused by the form of carbon source, which is mainly starch that is less available for hydrolysis, than soluble glucose in MRS broth [53,55,56,57].

One of the research limitations was a lack of analysis of metabolites produced by *Lactobacillus* spp. in the SSF medium. This should be studied in the future for further confirmation of its applicability as the cost-efficient medium for these bacteria cultivation. Moreover, it could unambiguously justify differences in the pH reduction of the newly developed medium which could be caused by variations in the type and amount of produced metabolites. Furthermore, assays were performed on a limited number of strains that probiotic properties were recently described [24]. Despite, it was sufficient to draw conclusions, the impact of the SSF medium on the growth performance of other *Lactobacillus* strains should be further investigated.

## 5. Conclusions

The new cost-efficient medium was composed based on the commercial flours from cereals, such as wheat, barley, maize, and rye, mixed with distilled water. The preparation of the media would not bring additional costs, since the most substantial growth of selected *Lactobacillus* strains was supported in the media with unmodified pH (6.0) and in the temperature, which is most often applied during *Lactobacillus* spp. cultivation (37 °C). The growth kinetics indicates that adaptation of bacteria to a new medium is necessary; however, the growth of the strains is more efficient. Therefore, the newly developed semi-solid fermentation medium is a promising alternative for high cell density production of *Lactobacillus* spp.

## Figures and Tables

**Table 1:** Nutritional values of used flours (information obtained from the package of each product).

Type of Flour	Energy	Carbohydrates	Total Lipids	Protein	Dietary Fibre
	[kcal/100 g]	[g/100 g]			
Wheat	350	73.0	1.6	11.0	NA ^1^
Barley	376	71.2	4.0	11.9	NA
Maize	337	70.5	3.0	5.6	7.5
Rye	301	74.0	2.2	8.1	13.0

^1^ not available.

**Table 2:** Viable cells productivity of selected *Lactobacillus* strains cultivated in SSF mediums with different water contents.

*Lactobacillus* Strain	Flours to Water Ratios		
	1:1.0	1:1.5	1:2.0
	VCP [× 10^9^ CFU/(kg × h)] ± SD ^1,2,3^		
*rhamnosus* ŁOCK 1087 ^4^	9.27 ± 1.23 ^a,5^	30.56 ± 6.36 ^b^	9.44 ± 2.66 ^a^
*paracasei* ŁOCK 1091	102.08 ± 13.66 ^a^	206.94 ± 36.22 ^b^	201.39 ± 24.85 ^b^
*reuteri* ŁOCK 1092	34.72 ± 3.18 ^a^	254.86 ± 37.81 ^c^	100.69 ± 10.28 ^b^
*plantarum* ŁOCK 0860	108.33 ± 16.54 ^a^	597.22 ± 55.68 ^c^	185.42 ± 29.39 ^b^
*pentosus* ŁOCK 1094	27.78 ± 2.41 ^a^	209.72 ± 16.84 ^c^	106.94 ± 20.32 ^b^

^1^ VCP stands for viable cells productivity; ^2^ CFU is an abbreviation of colony-forming units; ^3^ SD corresponds to a standard deviation; ^4^ ŁOCK is a designation of strains obtained from the Lodz Collection of Pure Cultures (Institute of Fermentation Technology and Microbiology, Lodz University of Technology; Lodz, Poland); ^5^ Various lowercase letters (a–c) represent significantly different mean values of VCP per one strain according to performed one-way ANOVA with post hoc Tukey’s test (*p* > 0.05).

**Table 3:** Growth parameters of selected *Lactobacillus* spp. probiotic strains cultivated in MRS and the SFF medium.

Parameter ^1^	µ_max_ [h^−1^]		λ [h]		GT [h]	
	MRS ^2^	SSF ^3^	MRS	SSF	MRS	SSF
*Lb. rhamnosus* ŁOCK 1087 ^4,5^	0.12	0.21	-	1.98	2.61	1.41
*Lb. paracasei* ŁOCK 1091	0.18	0.27	1.14	4.04	1.65	1.10
*Lb. reuteri* ŁOCK 1092	0.14	0.31	-	5.64	2.17	0.96
*Lb. plantarum* ŁOCK 0860	0.15	0.22	-	-	2.06	1.37
*Lb. pentosus* ŁOCK 1094	0.23	0.37	-	4.73	1.32	0.81

^1^ Analysed parameters: µ_max_—maximum growth rate; λ—duration of the lag phase; GT—generation time; ^2^ MRS is the abbreviation of de Man, Rogosa, and Sharpe broth; ^3^ SSF stand for the semi-solid fermentation medium; ^4^ *Lb.* is the abbreviation of *Lactobacillus* sp.; ^5^ ŁOCK is a designation of strains obtained from the Lodz Collection of Pure Cultures (Institute of Fermentation Technology and Microbiology, Lodz University of Technology; Lodz, Poland).
